# Prion Strains and Amyloid Polymorphism Influence Phenotypic Variation

**DOI:** 10.1371/journal.ppat.1004328

**Published:** 2014-09-04

**Authors:** Kevin C. Stein, Heather L. True

**Affiliations:** Department of Cell Biology and Physiology, Washington University School of Medicine, St. Louis, Missouri, United States of America; Duke University Medical Center, United States of America

## Introduction

The deposition of protein aggregates is a unifying feature of a large class of diseases known as protein conformational disorders, which includes Alzheimer disease and prion diseases. One of the most fascinating and puzzling aspects of such diseases is the phenomenon of amyloid polymorphism, whereby a single disease-associated protein forms different types of ordered aggregate structures. This is best exemplified in prion diseases, in which these different structures, called prion strains, are responsible for much of the variation in pathology and disease transmission. Here, we review the current knowledge of prion strains and amyloid polymorphism, highlighting how diversity in amyloid structure relates to phenotypic differences.

## Mammalian Prion Strains Dictate Differential Pathological Consequences

Like other fatal human neurodegenerative diseases, transmissible spongiform encephalopathies (TSEs), or prion diseases, have cases that arise sporadically (Creutzfeldt-Jakob disease [CJD]) or are inherited (fatal familial insomnia and Gerstmann-Sträussler-Scheinker syndrome) [1]. Remarkably, prion diseases can also be acquired by infection (e.g., kuru in humans). TSEs afflict a wide variety of mammalian species (e.g., scrapie in sheep, chronic wasting disease in cervids, and bovine spongiform encephalopathy [BSE] in cattle). These disorders are caused by conversion of the normal, host-encoded protein, PrP^C^, to an abnormal infectious conformation called PrP^Sc^ that generally adopts an amyloid-like structure. The widely accepted prion hypothesis suggests that PrP^Sc^ is the sole transmissible agent of prion diseases, thus making it distinct from conventional pathogens having a nucleic acid component. However, it was long unclear how a protein-based infectious agent could explain the existence of prion strains.

Even early observations of prion diseases describe considerable diversity in disease symptoms with different PrP^Sc^ isolates [2]. This typically presents as variation in incubation period (the time from infection to the onset of symptoms) or the distribution patterns of PrP^Sc^ or spongiform pathology in the brain. In addition, certain prion isolates show different degrees of transmissibility between species, a phenomenon called the “species barrier,” whereby transmission between different species is generally less efficient than transmission within the same species. This was brought to the public's attention in the mid-1990s with the outbreak of BSE (commonly referred to as “mad cow disease”) and subsequent transmission to humans, causing a novel disease called variant CJD [2]. Some argued that the variation in pathology and transmissibility related to prion strains indicated that the infectious agent must have a nucleic acid component or be encoded by changes in the PrP sequence in an analogous fashion as genetic polymorphisms that distinguish different strains of viral or bacterial infections. However, distinct prion strains were isolated that had an identical primary structure, suggesting that the physical basis of prion strains was not simply determined by sequence variation. Indeed, these early studies demonstrated that two different strains of transmissible mink encephalopathy showed different resistance to proteases, suggesting that prion strains represent distinct aggregate conformations of the same protein [3].

## PrP^Sc^ Strains Encode Variation in Structural Properties of Amyloid

In order to form distinct strains of PrP^Sc^, PrP^C^ undergoes a dramatic conformational change. PrP^C^ consists of an unstructured N-terminal domain and a C-terminal domain comprised of three α helices and two short β strands, making this structure ∼40% α helix and ∼5% β sheet [4]. However, formation of PrP^Sc^ is suggested to involve the conversion of the C-terminal domain into a β-sheet-rich amyloid-like structure (∼20% α helix and ∼43% β sheet), without any of the native α helices remaining [4]. However, this conformational change remains poorly understood.

While PrP^Sc^ strains exhibit amyloid polymorphism and many other disease-related proteins also form amyloid-like structures, there are a number of properties that are common to these ordered aggregates. Amyloid fibrils are generally unbranched structures that are 5–15 nm in diameter and are often comprised of multiple entwined protofilaments [5]. There are two generic amyloid-like folds. The most common is a cross-β sheet structure with β sheets that run parallel to the fibril axis, and individual β strands that form the β sheets are oriented perpendicular to the fibril axis [6]. The β strands can run in the same (parallel) or opposite (antiparallel) direction, with the parallel orientation generally being in-register, whereby each strand aligns with the identical residue in the neighboring monomer of the β sheet [6]. The common motif formed by the β sheets is called a steric zipper, in which the side chains of opposing β sheets interdigitate using hydrogen bonds or van der Waals interactions to form a complementary interface that is free from exposure to solvent [7]. Such a strong underlying hydrophobic effect helps make amyloid a very stable structure [6].

By contrast, while there is still considerable debate, PrP^Sc^ is proposed to form the second generic amyloid-like fold called a β solenoid [4]. Here, a single monomer will form three β strands that loop around each other in a helix-like structure [5]. Although it remains technically challenging to decipher many additional details about the structure of PrP^Sc^, a number of observations indicate certain variables that might contribute to the structural differences of prion strains: (1) the length of the region protected in β sheets, as well as what sequence is protected [8]; (2) whether the protein is truncated, as the C-terminally truncated Y145Stop construct of human PrP has a very different region of the primary structure that forms the amyloid core as compared to longer versions of the protein [7] (and different truncated versions of PrP^Sc^ have been isolated from brains [1]); and (3) sensitivity to protease digestion, with both protease-resistant and sensitive forms of PrP^Sc^ existing [9]. However, the extent to which these factors contribute to amyloid polymorphism is unclear, and moreover, the connection of amyloid polymorphism to pathological variation remains elusive.

## Biophysical Parameters Define Strains of the Yeast Prion [*PSI*+]

Significant insight into how prion strains can mediate phenotypic differences has come from studying the endogenous prion proteins that exist in the budding yeast, *Saccharomyces cerevisiae* [10]. The [*PSI*+] prion is formed from the translation termination factor Sup35, which can form self-propagating aggregates. Sequestration of Sup35 into the prion state impairs translation termination, causing ribosomes to read through stop codons (nonsense suppression). Strains of [*PSI*+] (called variants in yeast, but for simplicity, we will use the term strains) are characterized by the degree of nonsense suppression: cells propagating a strong [*PSI*+] strain exhibit greater nonsense suppression as compared to weak [*PSI*+] cells [11].

A model describing the structural basis of these strain-dependent phenotypes has served as the foundation for understanding prion strains [12]. This model suggests that a set of biophysical parameters defines the nature of the prion strain that propagates. Structurally, these parameters are dictated by the length of the amyloid core, that is, the number of amino acid residues that are protected in β sheets [13]. An expansion of the core, by incorporating more residues into hydrogen-bonded β sheets, as in the case of weak [*PSI*+], correlated with an increase in aggregate stability [12], [13]. It was reasoned that higher stability decreased how readily the amyloid could be fragmented, thus resulting in seeds that are fewer in number and have a larger average size. Consequently, fewer surfaces are available to recruit monomeric Sup35 in cells harboring weak [*PSI*+]. This leads to a larger pool of soluble Sup35 to function in translation termination (i.e., less nonsense suppression) as compared to strong [*PSI*+] cells that have aggregates that sequester more Sup35 monomer. Hence, aggregate stability and amyloid core length were suggested to be the major determinants of strain-dependent phenotypes [12], [13].

This same correlation was found to fit with some synthetic prion strains of PrP^Sc^: decreased aggregate stability correlated with a shorter incubation period of disease [14]. However, several recent examples of other PrP^Sc^ strains cannot be explained by the biophysical parameters established by the model of [*PSI*+] strains: (1) strains with different stability had a similar core length [15]; (2) decreased stability of other PrP^Sc^ strains correlated with a longer disease incubation time [16]; and (3) strains that are biochemically indistinguishable can confer distinct pathological consequences [17]. This exemplifies the wide variety of different amyloid-like structures that a single polypeptide can form, leaving it unclear how other factors might influence strain-mediated phenotypic variation.

## Differential Chaperone Interactions and Amyloidogenic Regions Influence the Complex Nature of [*RNQ*+] Strains

Another yeast prion called [*RNQ*+], which is formed from the Rnq1 protein, manifests phenotypically by promoting the formation of [*PSI*+] [18]. Different prion strains of [*RNQ*+] are characterized by how readily [*PSI*+] forms. The Rnq1 protein was found to form a remarkable variety of structural variants that exhibit tremendous variation in the ability to promote [*PSI*+] induction [18], [19]. As with Sup35 in [*PSI*+] cells, aggregate stability was shown to be a defining factor in the propagation of particular [*RNQ*+] strains [20]. However, it was recently found that aggregate stability, along with several other biophysical properties that distinguish strains of [*PSI*+], were unable to distinguish other [*RNQ*+] strains [21], [22].

**Figure 1:**
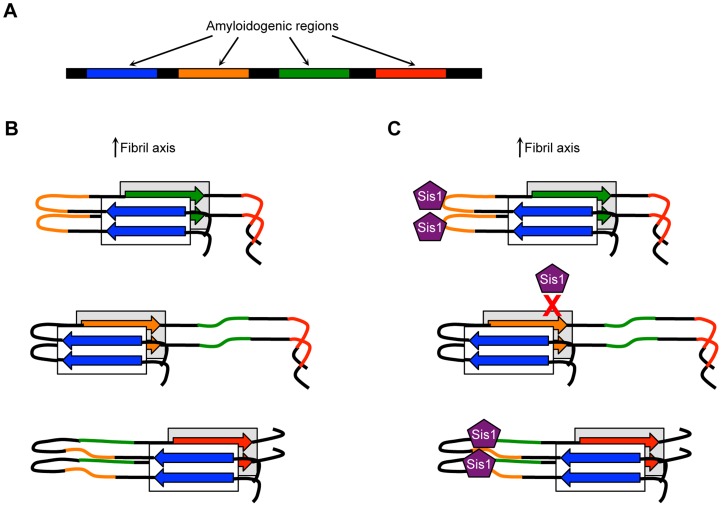
Model showing how distinct amyloidogenic regions could influence amyloid polymorphism and associated phenotypic variation. (A) A single protein can have multiple amyloidogenic regions (colored as blue, orange, green, and red) that are not adjacent in the primary structure. (B) These regions can influence amyloid packing in a variety of ways, with nonadjacent regions possibly forming the amyloid core. (C) If a particular amyloidogenic region represents a chaperone-binding site (e.g., the Hsp40 Sis1 has affinity for the orange region), this region is exposed and available for binding in certain structures (top and bottom) but not others (middle). In addition, chaperone or cofactor binding prior to amyloid folding might influence the range of amyloid structures that can form or propagate, thereby providing a mechanism by which genetic and environmental modifiers might alter amyloid structure.

Mutation analysis of the Rnq1 protein with five different [*RNQ*+] strains revealed additional factors that can contribute to amyloid polymorphism and phenotypic differences. Multiple regions predicted to be amyloidogenic were identified throughout the Rnq1 protein [21]. These regions are postulated to influence the formation of amyloid [23]. It was found that the propagation and phenotypic variation (i.e., [*PSI*+] induction) of each [*RNQ*+] strain relied on a distinct set of nonadjacent amyloidogenic regions (Figure 1A, 1B) [21], in stark contrast to [*PSI*+] strains that have a contiguous region of Sup35 protected in the amyloid core [13]. Moreover, both Sup35 and Rnq1 have prion-forming domains (PFD) that are rich in glutamine and asparagine (Q/N) residues and are necessary for prion propagation [24]. However, it was shown that the region outside of the Rnq1-PFD played a major, but strain-dependent, role in prion propagation by facilitating sequestration of monomer into aggregates, thereby highlighting the influence of noncanonical regions on prion strains.

In addition to the differential influence of primary structure, it was found that [*RNQ*+] strains likely have diverse interactions with molecular chaperones [21]. From yeast to humans, molecular chaperones are involved in processing misfolded and aggregated proteins. Such processing, in the case of yeast prions, is required for the continued maintenance of the prion state [10]. A peptide-binding array identified one of the Rnq1 amyloidogenic regions as important for Sis1 binding, which is a required component of prion propagation [25]. However, in another study, it was shown that Sis1 could bind other regions of Rnq1 [21]. Indeed, a distinct amyloidogenic region was particularly important for the propagation of one [*RNQ*+] strain and might serve as a second Sis1 binding site. This suggests that conformation could dictate the exposure of different binding sites and/or the affinity of chaperones for the same site (Figure 1C). Moreover, chaperone binding to a specific site at an early stage of folding may influence the amyloid structure that forms. These differences are also likely to be true for [*PSI*+] strains [26] and agree with the hypothesis that PrP^Sc^ strains have different interactions with cofactors [27]. Additionally, this provides insight into how changes in extracellular environment may mediate prion strain generation and propagation [28], [29]. Hence, variation in amyloid-chaperone sites of interaction is likely a major determinant of the phenotypic differences caused by prion strains.

## Amyloid Polymorphism Is a Ubiquitous Feature of Disease-Associated Proteins

In recent years, the prevalence of amyloid polymorphism has been extended to many different proteins associated with protein conformational disorders. For instance, based on histopathology and biochemical properties, amyloid-beta (Aβ) forms heterogeneous deposits in the brains of patients with Alzheimer disease [6]. Heterogeneity was also observed in vivo for other proteins, including tau, α-synuclein, and transthyretin, suggesting that the phenomenon of amyloid polymorphism is widespread and not limited to prion proteins [6].

Amyloid polymorphism has also been extensively studied using synthetic polypeptides [30]–[32]. It was demonstrated that the same sequence could form multiple different steric zipper structures, which were proposed to fall into eight different classes [31]. Furthermore, three different models of polymorphism were postulated to explain the diversity of structures that were observed: packing (different β sheet arrangements of the same sequence), segmental (different sequences form similar β sheet conformation), and heterozipper (different regions that are cross complementary form the β sheet, either two regions in the same monomer or between two monomers) [32]. When considering these data in the context of a full-length protein, which can have several amyloidogenic regions, combined with different cofactor requirements [27], it then becomes easier to envision a considerable number of stable amyloid structures that are theoretically possible. Indeed, one conformation being uniquely thermodynamically stable above all other combinations seems unrealistic simply in terms of probability. Therefore, elucidating the complex interplay of variables that affect the formation and maintenance of polymorphic structures remains a nontrivial, even crucial, task to gain a full understanding of pathological variability and the etiology of protein conformational disorders.
